# A Stability Atlas for IBSI Radiomics Features Using Synthetic Digital Phantoms, with Proof-of-Concept Physics-Based Normalisation

**DOI:** 10.3390/jimaging12080392

**Published:** 2026-08-20

**Authors:** Shuji Yamamoto

**Affiliations:** Institute of One, LISIT Co., Ltd., Tokyo 150-0044, Japan; yamamoto@lisit.jp

**Keywords:** radiomics, reproducibility, IBSI, digital phantom, feature stability, image acquisition, open source

## Abstract

Radiomics features are strongly sensitive to image acquisition, and separating that sensitivity from biological signal usually requires repeated patient scans that cannot be shared. We present an open, fully synthetic framework (radiomics-phantom) that maps and, as a proof of concept, corrects radiomics feature instability without any patient data. Deterministic three-dimensional texture phantoms are generated as anisotropic Gaussian random fields with known ground truth and an optional embedded lesion. An independently implemented feature core aligned with the Image Biomarker Standardization Initiative (IBSI) covers all eleven IBSI-1 feature families and matched all 482 published digital-phantom benchmark values within the applicable tolerances. An image-domain acquisition simulator applies point-spread blur, slice-profile averaging, dose-scaled correlated noise, resampling, and quantisation. Per-feature reproducibility across a sweep of fifteen textures (varying correlation length, anisotropy, and intensity scale) by nine acquisition conditions, with five independent noise realisations per stochastic setting, is summarised by the absolute-agreement intraclass correlation ICC(2,1), with a realisation-aware percentile-bootstrap 95% confidence interval for every estimate; constant features are excluded from estimation. Values span nearly the full range (median 0.13, 95% CI 0.03–0.19), and a hierarchical variance decomposition attributes a median 77% of per-feature variance to the acquisition condition and under 1% to stochastic realisation; the values are interpreted as exploratory rankings within this acquisition envelope. As a proof of concept, intensity variance and grey-level co-occurrence contrast under additive Gaussian noise were normalised using calibrated, invertible response models, returning them to their noiseless values on held-out data (median error below 4% across five textures and repeated noise realisations, and about 11% when the noise level is estimated from the degraded image itself), while features the models cannot describe are refused rather than corrected. All code and a 716-test suite are released openly and archived on Zenodo. The result is a reproducible, patient-data-free testbed for radiomics feature stability.

## 1. Introduction

Radiomics extracts large numbers of quantitative features from medical images to characterise tissue phenotypes [1]. A persistent obstacle to clinical translation is that many features are highly sensitive to how the image was acquired and reconstructed—dose, reconstruction kernel, slice thickness, and voxel size—so that measured values reflect the scanner as much as the biology; phantom and multi-parameter studies report that a large fraction of CT radiomic features are non-reproducible under acquisition change [2,3]. The Image Biomarker Standardization Initiative (IBSI) has standardised feature definitions and provides a digital reference phantom with benchmark values [4], and widely used toolkits implement these definitions [5]. Standardised *definitions*, however, do not make feature *values* reproducible across acquisitions by themselves, and residual differences persist even among standards-aligned implementations [6].

Studying this reproducibility normally requires repeated patient scans, which are scarce and cannot be shared, or post hoc harmonisation against a reference cohort (for example ComBat [7,8]), which treats acquisition effects as a nuisance to be regressed out rather than as known physics. We take a different route: a fully synthetic testbed in which the texture is known by construction and the acquisition is a controlled, image-domain transformation. Because nothing derives from a human subject, the entire study is shareable and, for a fixed software environment, reproducible.

This work provides (a) an open, deterministic generator of 3D texture phantoms with ground truth, paired with an independently implemented, IBSI-aligned feature core benchmarked against the full set of published digital-phantom values; (b) a *stability atlas* that ranks every feature’s reproducibility across a texture-by-acquisition sweep using ICC(2,1) with bootstrap 95% confidence intervals (with the concordance correlation coefficient as a secondary metric); and (c) a proof-of-concept, physics-based normalisation that corrects features (intensity variance and grey-level co-occurrence contrast) using their known or empirically calibrated responses to a measurable acquisition descriptor and refuses features whose response it cannot model.

The framework occupies a niche that, to our knowledge, no existing tool covers, and Section 4.2 positions it against each neighbouring tool class in detail: feature-extraction toolkits validate definitions but generate neither ground-truth phantoms nor acquisition sweeps [5]; the IBSI digital phantom is a fixed validation reference rather than a configurable testbed [4]; physical texture phantoms provide real scanner effects but no parametric ground truth and no shareable raw data [3]; projection-domain simulation chains offer far higher physical fidelity at much higher cost and complexity [9,10]; and cohort-based harmonisation corrects distributions statistically without a generative acquisition model [7,8]. The work is therefore positioned, throughout, as an integrated, reproducible research framework rather than a new radiomics methodology: each ingredient has precedents, and the claimed contribution is the combination—parametric ground truth, a benchmarked independent feature core, a controlled acquisition sweep, reliability screening with uncertainty, and refusal-based normalisation—in one open, deterministic, pure-Python package.

## 2. Materials and Methods

**Figure 1:**
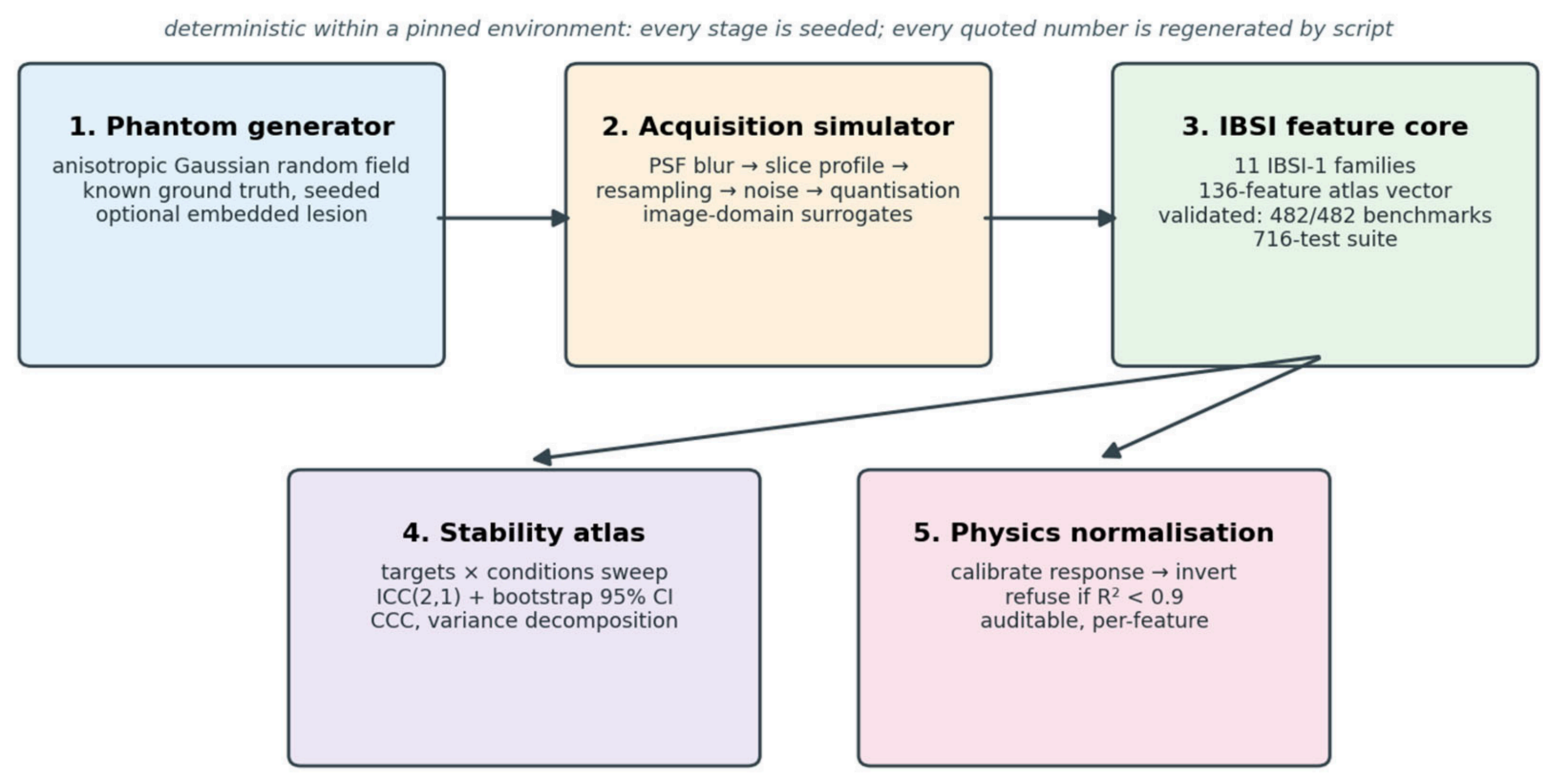
Overview of the radiomics-phantom framework and its five stages; every stage is seeded and every quoted number is regenerated by script.

Figure 1 gives an overview of the framework: a deterministic phantom generator (Section 2.1) feeds an image-domain acquisition simulator (Section 2.3); the benchmarked feature core (Section 2.2) measures every simulated acquisition; and the resulting feature tables drive the stability atlas (Section 2.4) and the physics-based normalisation (Section 2.5).

### 2.1. Synthetic Texture Phantom

The background parenchyma is a stationary Gaussian random field: white noise convolved with an anisotropic Gaussian kernel in the Fourier domain. A kernel of standard deviation *s* produces an autocovariance $exp\left(-r^{2}/4s^{2}\right)$, so the lag at which correlation falls to 1/e is 2s; that lag, in millimetres, is the prescribed correlation length, controllable per axis to yield anisotropic texture. An optional ellipsoidal lesion is filled with a second, independent field of different mean, contrast, and correlation length. Every generator takes a seed and, within a fixed software environment, returns the same volume together with its ground truth. An empirical estimator recovers the correlation length from the power spectrum, confirming that the field carries the requested texture.

### 2.2. IBSI-Aligned Feature Core

All eleven IBSI-1 families are implemented from their definitions using only array primitives: intensity statistics and histogram, the intensity–volume histogram, morphology and local intensity, and the six texture families (grey-level co-occurrence, run length, size zone, distance zone, neighbourhood grey-tone difference, and neighbouring grey-level dependence), each over their IBSI aggregations. Fixed-bin-size discretisation is used as the default for calibrated HU-like intensities in this framework; IBSI also defines and benchmarks fixed-bin-number configurations. Degenerate regions raise explicit errors rather than returning undefined values. This benchmark validates the tested feature definitions and aggregation settings; it does not establish compliance across every IBSI preprocessing configuration by itself.

### 2.3. Acquisition Simulator

The same texture is observed under many simulated scanners by applying deterministic, image-domain surrogates for the dominant acquisition effects: a Gaussian point-spread function for in-plane spatial resolution loss (specified by full width at half maximum), through-plane averaging for the slice profile, correlated Gaussian noise with dose-dependent amplitude (scaled as $1/\sqrt{\mathrm{dose}}$) optionally coloured like a reconstruction kernel, interpolation onto a different voxel grid, and intensity quantisation. It is not a projection-domain CT reconstruction simulator; the resulting limits on generalisability are examined in Section 4.3. As implemented, the in-plane point-spread blur and the through-plane slice-profile averaging are applied together as one anisotropic Gaussian, in the order: spatial resolution and slice-profile blur, then resampling, then noise, then quantisation. Only noise is stochastic and is seeded; for a fixed environment, a given phantom, settings, and seed give the same acquisition.

### 2.4. Stability-Atlas Design

A set of phantom textures (the *targets*) is observed under a set of acquisition settings (the *conditions*). The atlas reported here uses fifteen targets ($32^{3}$ voxels of 1 mm), spanning five background correlation lengths (3–9 mm), three anisotropy settings (isotropic, 2× along one axis, 1.5× along two axes), and two intensity-scale variants (background standard deviation 15 and 35 in place of the default 25); and nine conditions: a noiseless reference, three noise levels (σ = 7.5, 15, 30), two blur levels (FWHM 1.5 and 3 mm), a 3 mm slice profile with resampling to 2 mm slices, quantisation (step 10) with mild noise, and a combined noise-plus-blur setting. For each feature, reproducibility is summarised by the two-way random-effects, single-measurement, absolute-agreement intraclass correlation ICC(2,1) [11] across the target-by-condition table, with the choice and interpretation of ICC form following established guidance [12]. ICC(2,1) is used as an absolute-agreement measure across acquisition conditions treated as a sampled acquisition envelope. Lin’s concordance correlation coefficient [13] of each degraded condition against the reference is reported as a secondary metric (supplementary table). Both statistics are implemented from first principles and cross-checked against an independent library (pingouin) in the test suite.

Features that are constant across the entire sweep carry no reliability information, and applying the ICC estimator to zero-variance data yields a definitional artefact rather than measured agreement; such features are therefore identified and excluded from reliability estimation, and listed separately in the supplementary table. Sampling uncertainty is quantified by a percentile bootstrap over targets (2000 replicates with draws shared across features): a 95% confidence interval accompanies every per-feature ICC and the median ICC, and rank stability is assessed as the Spearman correlation between the point-estimate feature ranking and each bootstrap replicate’s ranking. The two-way ANOVA underlying the ICC additionally yields, per feature, a decomposition of variance into target, condition, and residual components, reported as fractions of the total. The atlas performs no hypothesis tests and applies no significance thresholds—it reports estimates with uncertainty, not decisions—so no multiple-comparison correction is applicable.

Stochastic acquisition effects are represented by five independent noise realisations for every target–condition pair whose condition includes noise (five of the nine conditions; 435 simulated acquisitions in total); deterministic conditions have no stochastic component by construction. Reliability statistics are computed on per-cell means over realisations, and the percentile bootstrap is realisation-aware: each replicate resamples both the targets and, within every stochastic cell, the noise realisations, so the confidence intervals reflect texture sampling and stochastic acquisition noise jointly. The two-way analysis is extended to a hierarchical variance decomposition that separates target (texture), condition, target-by-condition interaction, and stochastic–realisation components, the last estimated from the pooled within-cell variance across realisations. Because the nine conditions are deliberately constructed rather than randomly sampled from a defined scanner population, the choice of ICC form deserves justification: ICC(2,1) is retained as the primary statistic because absolute agreement across the envelope—which penalises systematic condition-induced shifts—is precisely what the reproducibility of feature values requires, with the envelope read as the population the estimate generalises to. As a sensitivity analysis, the fixed-effects consistency form ICC(3,1) is reported for every feature, and the envelope’s compositional influence is quantified by recomputing every ICC under leave-one-condition-out.

### 2.5. Physics-Based Normalisation (Proof of Concept)

As a proof of concept, rather than harmonise against a cohort, a feature’s response to one acquisition descriptor is calibrated on the phantom by sweeping that descriptor and fitting a simple invertible model (linear or power); the model is then inverted to map an observed feature back to a reference acquisition. Calibration and evaluation use different noise realisations (separate seeds and held-out noise levels). A feature whose calibration the model cannot describe (coefficient of determination below 0.9) is refused rather than silently corrected. The proof of concept covers two feature–response pairs under additive Gaussian noise: intensity variance, whose response has a closed analytic form (independent zero-mean noise adds in quadrature), and grey-level co-occurrence (GLCM) contrast, whose response has no closed form here but is tested for empirical fit by the same quadratic model. We use *physics-based* strictly for the former case, where the response follows a closed physical law; the latter is more precisely a *physics-informed, empirically calibrated* normalisation, in which the model form is motivated by the noise physics but its validity rests on the measured calibration fit and the held-out evaluation, not on a derivation. This demonstrates the framework’s ability to calibrate and invert a known or empirically smooth physical response; it does not establish general normalisation for all radiomics features.

The proof of concept is validated beyond a single texture and seed. GLCM contrast is calibrated and evaluated on five textures with three held-out noise realisations each; correction error is reported with its distribution. A practical variant estimates the noise level from the degraded image itself (Immerkaer’s fast noise estimator [14], averaged over axial slices) instead of taking it from the simulator, so that the descriptor is measured rather than known. Three failure probes are included by design: out-of-range extrapolation beyond the calibrated interval; a confounded calibration in which noise and blur co-vary, which the goodness-of-fit screen cannot detect; and model misspecification, applying a white-noise calibration to correlated noise. Finally, the acceptance threshold is examined out-of-sample: for every feature with a measurable noise response, the calibration coefficient of determination is related to the held-out fraction of drift remaining after correction.

### 2.6. Implementation, Scope, and Reproducibility

The software is pure Python (numpy 2.4.4, scipy 1.17.1, scikit-image 0.26.0, matplotlib 3.10.9, and pingouin 0.6.1, with an optional tkinter GUI) and is deterministic within a pinned software environment for a fixed seed. It is covered by 716 automated tests; continuous integration tests verify numerical outputs against predefined tolerances across Python 3.10–3.12. It is released under the MIT licence (text and figures under CC BY 4.0) and archived on Zenodo. An interactive desktop tool (Phantom Studio) exposes the phantom, acquisition, and feature stages for exploration. The IBSI test fixtures are regenerated from their authoritative sources by a script (scripts/fetch_ibsi_reference.py); the figures, the supplementary tables, and every quoted number are produced by paper/make_figures.py, paper/make_figures_r1.py, paper/make_figures_r2.py, and paper/make_supplementary.py and written to paper/figures/ and paper/supplementary/ so that text, tables, and figures cannot diverge. The framework is computationally light: on a single commodity CPU core (Linux container, Python 3.11), generating one $32^{3}$ phantom takes about 3 ms, simulating one acquisition about 2 ms, and extracting the full 136-feature vector about 80 ms. The entire expanded atlas—135 simulated acquisitions with feature extraction, plus 2000 bootstrap replicates over 134 features—completes in roughly half a minute (machine-dependent timings are written to paper/figures/timing.json). Section 4.4 discusses computational cost further. This study reports the open core and its reproducible synthetic benchmarks; no clinical or real data validation is claimed.

### 2.7. Use of Generative AI

In accordance with MDPI policy, the author discloses that generative AI was used as a tool during this work. A large language model (Claude (Anthropic; principally Claude Fable 5, with early code development assisted by Claude Opus 4)) assisted with code scaffolding and refactoring of the software modules, test drafting, figure and script generation, and manuscript drafting. The author independently re-executed every numerical result reported here (the IBSI validation, the acquisition sweep, the stability atlas, the normalisation, and the test suite) and verified all figures, equations, and claims against the code. No AI system meets the criteria for authorship, and the author takes full responsibility for the content.

## 3. Results

### 3.1. IBSI Validation

**Figure 2:**
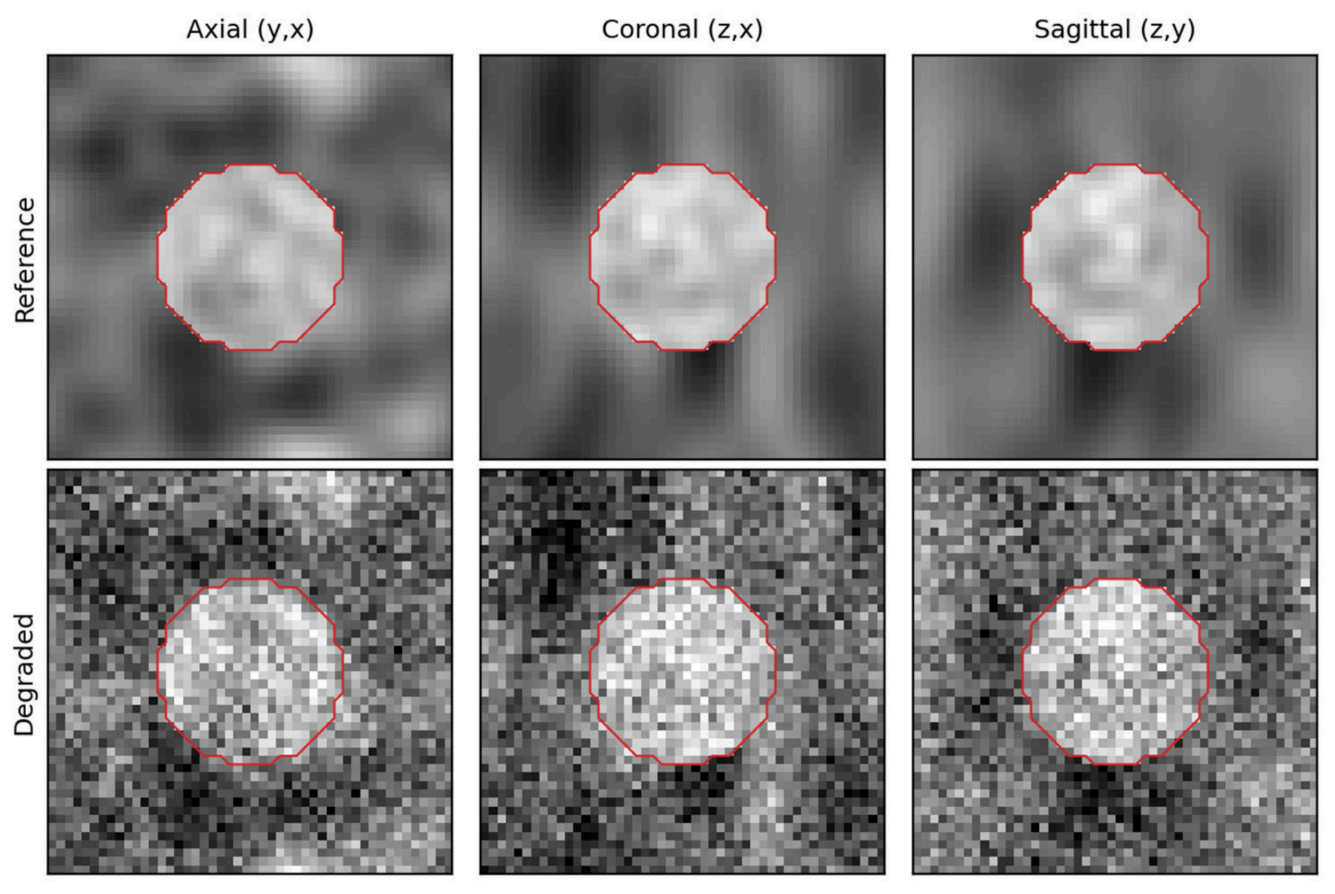
A synthetic phantom (**top**) and the same phantom under a simulated acquisition with reconstruction-kernel blur and additive noise (**bottom**), in three orthogonal planes; the red contour is the ground-truth lesion mask.

Computed on the IBSI digital phantom, the feature core matched all 482 published digital-phantom benchmark values at the reported precision (three significant figures) and within the applicable IBSI tolerances, across every implemented family and aggregation. This benchmark validates the tested feature definitions and aggregation settings; it does not by itself establish compliance across every IBSI preprocessing configuration. The four bounding-shape density features that IBSI leaves unstandardised are the only IBSI-1 features not implemented. Test fixtures are regenerated directly from the authoritative IBSI sources rather than transcribed, and the benchmark assertions run in continuous integration. A representative phantom and one simulated acquisition are shown in Figure 2.

The 482 benchmark values span all families and aggregation methods (for example, each texture feature is benchmarked under several aggregations). The stability atlas below uses a defined 136-feature vector: the 3D-merged aggregation for the directional texture families and the 3D aggregation for the zone, neighbourhood, and dependence families, together with intensity statistics and histogram, and excluding morphology, local intensity, and intensity–volume histogram features (which require a lesion mask and are not meaningful on the whole-volume region used here). Two of the 136 features are constant on the phantoms used here and are excluded from reliability estimation (Section 2.4), leaving 134 measured features. A machine-readable mapping from every benchmark value to its inclusion in the atlas is provided (paper/supplementary/feature_inventory.csv).

### 3.2. Acquisition Sensitivity

The degradations move features in the direction physics predicts. For a representative grey-level co-occurrence contrast feature on a fixed texture, adding noise of increasing standard deviation raised contrast monotonically (from 0.26 with no noise to 0.60, 2.28, and 8.27 at noise levels of 10, 25, and 50 HU-like units), while increasing reconstruction-kernel blur lowered it (from 0.26 to 0.24, 0.21, and 0.16 at full widths at half maximum of 2, 4, and 6 mm). Independent noise adds in quadrature to the intensity variance, as expected.

### 3.3. Stability Atlas

**Figure 3:**
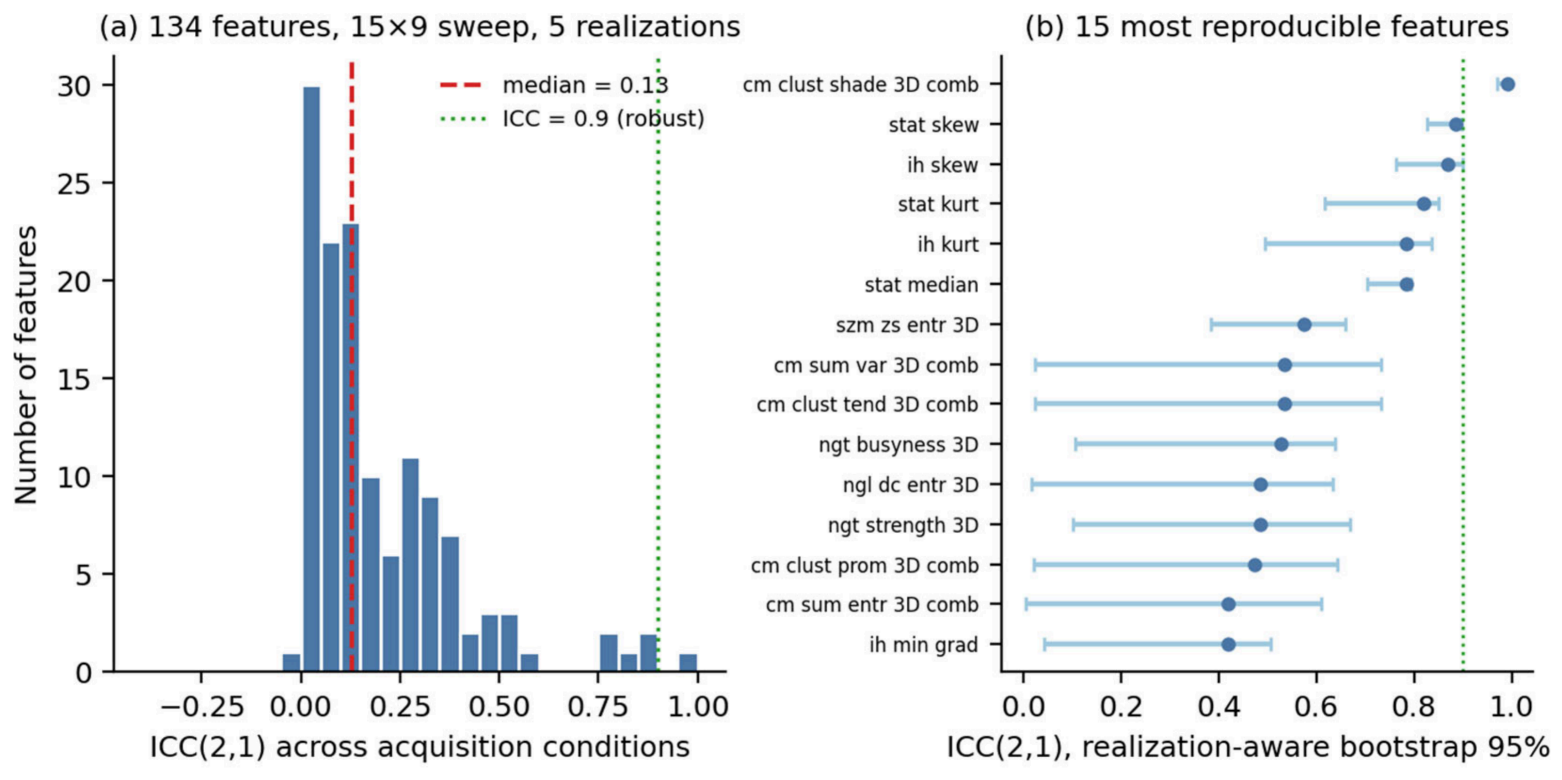
Stability atlas under the fifteen-by-nine sweep with five noise realisations per stochastic condition: (**a**) distribution of ICC(2,1) across the 134 measured features (dashed line: median; dotted line: 0.9 robustness threshold); (**b**) the fifteen most reproducible features with realisation-aware bootstrap 95% confidence intervals.

When sweeping fifteen phantom textures across nine acquisition conditions with five noise realisations per stochastic setting (Section 2.4) and rating the 134 measured features, reproducibility spanned nearly the full range of ICC (median 0.13, realisation-aware bootstrap 95% CI 0.03–0.19; maximum 0.99) under these deliberately harsh conditions (Figure 3a). The two constant features remain excluded from the estimation a priori. The median 95% interval width was 0.15 (interquartile range 0.07–0.36), essentially unchanged from the target-only bootstrap of the previous revision (0.14), because stochastic realisation contributes little variance for most features: the hierarchical decomposition attributes, per feature, a median 77% of variance to the acquisition condition, 12% to the texture target, 4% to the target-by-condition interaction, and 0.7% to stochastic realisation (maximum 38% for the most noise-sensitive features). Acquisition effects, not stochastic noise or texture, dominate this envelope.

Only one feature exceeded the conventional 0.9 robustness threshold: GLCM cluster shade (ICC 0.992, 95% CI 0.971–0.994), the only feature whose lower confidence bound exceeds 0.9. This apparent robustness is explicitly limited to the present synthetic acquisition envelope and carries no claim about clinical acquisitions. The next most reproducible were intensity skewness (0.885, CI 0.826–0.896), histogram skewness (0.870, CI 0.763–0.900), intensity kurtosis (0.820, CI 0.617–0.851), histogram kurtosis (0.784, CI 0.494–0.836), and median intensity (0.783, CI 0.704–0.794). The fixed-effects sensitivity analysis illuminates what the choice of ICC form measures: ICC(3,1) agrees with ICC(2,1) for the skewness-family features at the top of the ranking, but rates features with large systematic condition shifts far higher (for example, GLCM sum variance: ICC(2,1) = 0.54 versus ICC(3,1) = 0.98, Spearman correlation between the two rankings 0.55 over all features), because consistency ignores exactly the condition-induced level shifts that absolute agreement penalises—supporting ICC(2,1) as the primary reproducibility measure. The envelope composition analysis shows that removing any single condition changes a typical feature’s ICC by a median range of 0.11 (IQR 0.06–0.15), while the top-ranked feature is essentially envelope-stable (range 0.003). Mid-ranking positions are therefore sensitive to the envelope’s composition—another reason the atlas is an exploratory screen. Feature rankings were broadly stable under the realisation-aware bootstrap (mean Spearman’s rho = 0.90 against the point-estimate ranking, 95% CI 0.37–0.99). Per-feature ICC(2,1) with confidence intervals, ICC(3,1), leave-one-condition-out range, and the four-component variance fractions are provided (paper/supplementary/stability_atlas.csv).

### 3.4. Physics-Based Normalisation

**Figure 4:**
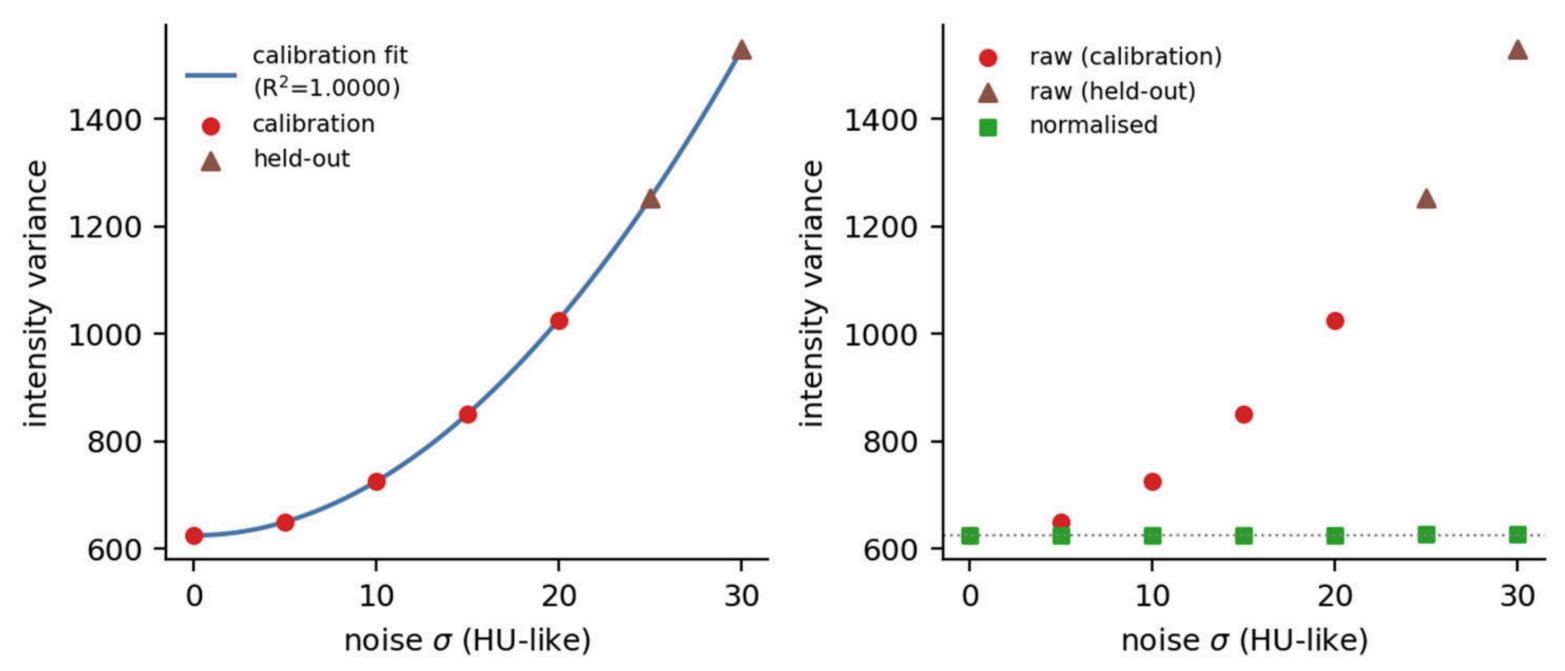
Proof-of-concept physics-based normalisation of intensity variance: the fitted quadratic variance–noise model (**left**) and raw versus normalised variance on held-out noise levels (**right**); the dotted line marks the noiseless variance.

As a proof of concept, intensity variance was normalised under additive Gaussian noise using the analytic relationship between signal variance and known noise variance: independent zero-mean noise adds in quadrature, so variance follows ${var}_{0}+b\;\sigma^{2}$. Calibrating on noise levels 0–20 (one noise realisation) gave a fitted slope b=1.00 and intercept equal to the noiseless variance (625 HU-like units squared), at $R^{2}=1.000$ (Figure 4, left). Evaluated on held-out noise levels (25 and 30) generated with a different seed, the raw variance rose to 1254 and 1529, and inverting the calibration returned it to 628 and 628—within about three units of the noiseless value of 625 (Figure 4, right). While this validates the framework’s ability to calibrate and invert a known physical response, it does not establish general normalisation for all radiomics features. Consistently, features whose response the power-2 model cannot describe are refused; for example, mean intensity, which has no variance-like response to zero-mean noise (fitted $R^{2}=0.19$, below the 0.9 acceptance threshold), was correctly rejected rather than corrected.

**Figure 5:**
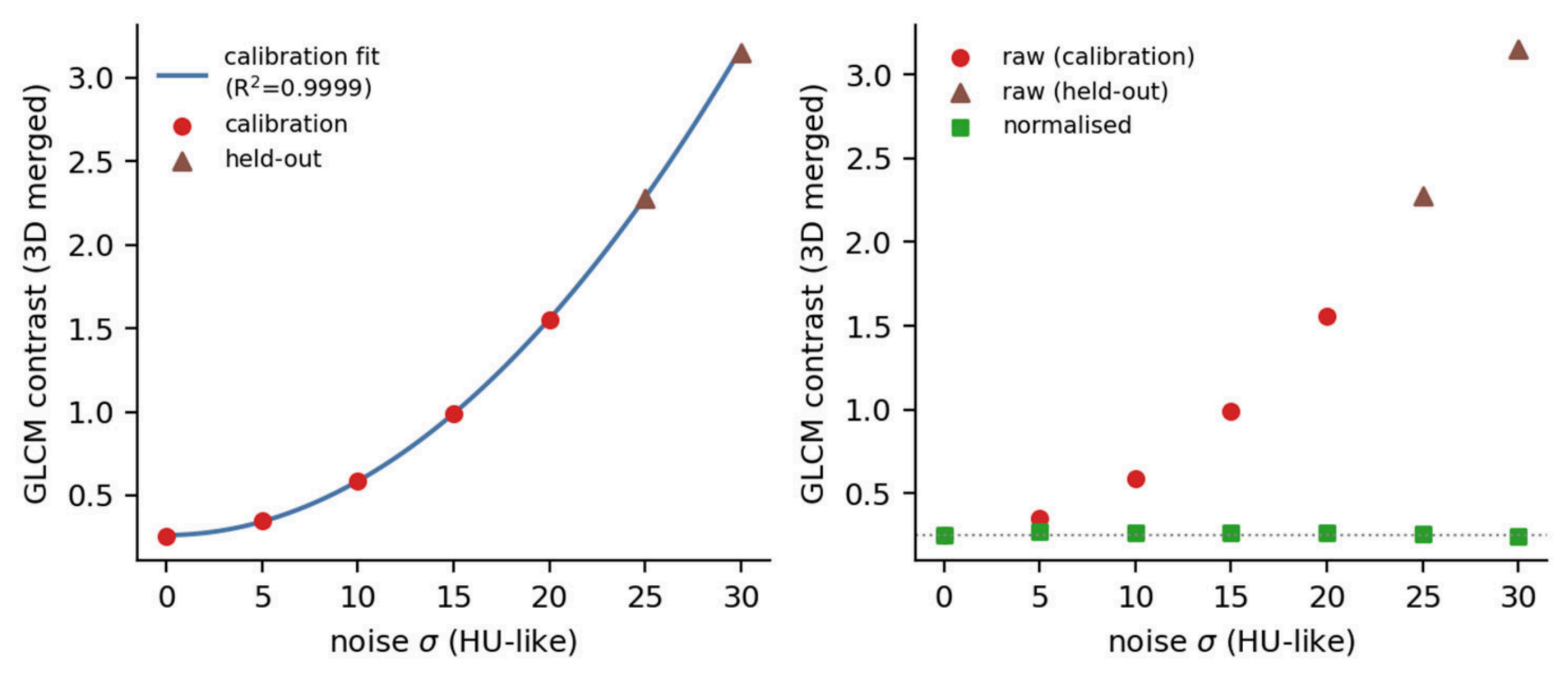
Physics-informed, empirically calibrated normalisation of GLCM contrast (3D merged) under additive Gaussian noise: calibration fit (**left**) and raw versus normalised contrast on held-out noise levels (**right**); the dotted line marks the noiseless value.

The same calibrate-and-invert protocol was then applied to a higher-order texture feature: GLCM contrast (3D-merged aggregation), whose response to additive noise has no closed analytic form in this setting. Calibrating the quadratic model on noise levels 0–20 gave b=0.0032 per squared noise unit at $R^{2}=0.9999$; the empirical response is almost exactly quadratic, as expected qualitatively because uncorrelated noise inflates every co-occurrence intensity difference. On the held-out noise levels (25 and 30, different seed), the raw contrast rose from its noiseless value of 0.254 to 2.28 and 3.15—a nine- to twelve-fold drift—and inverting the calibration returned it to 0.256 and 0.242, within 1.0% and 4.6% of the noiseless value (Figure 5). A higher-order texture feature can therefore be corrected by the same route—here better described as physics-informed and empirically calibrated (Section 2.5)—when its measured response to the descriptor is smooth and invertible within the calibrated range. The residual error is larger than for intensity variance because here the quadratic model is empirical rather than exact. Features whose responses are non-monotonic, non-smooth, or dependent on more than one descriptor will not pass the acceptance threshold and are refused; the scope of this mechanism is discussed in Section 4.3.

**Figure 6:**
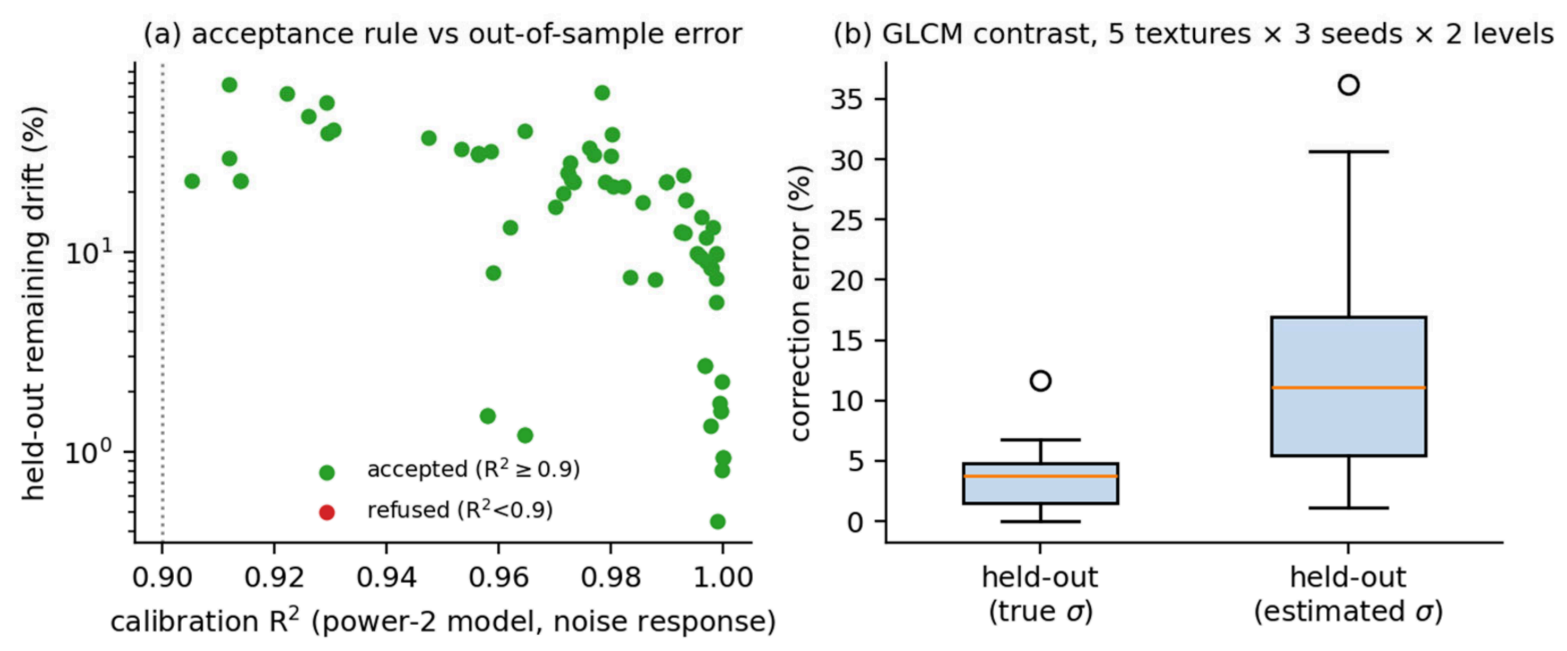
Out-of-sample validation of the normalisation: (**a**) calibration fit versus held-out remaining drift across 75 feature–response pairs; (**b**) GLCM contrast correction error over five textures and three held-out noise realisations, with true versus image-estimated noise level.

The proof of concept was then stress-tested (Figure 6). Across five textures with three held-out noise realisations each (30 evaluations), correction with the true noise level left a median error of 3.8% (95th percentile 6.7%) relative to the noiseless value. Estimating the noise level from the degraded image itself (median estimation error 0.6%) raised the median correction error to 11.1% (95th percentile 29.6%). Because the raw drift at these levels is nine- to twelvefold, the quadratic response amplifies even sub-percent errors in the estimated descriptor; the estimated descriptor correction still removes roughly 98–99% of the drift. Extrapolating outside the calibrated range (calibrated at noise 0–20, applied at 40 and 50) left errors of 9.0% and 4.5%. Three failure modes were probed explicitly. First, a feature whose response the model cannot describe is refused: mean intensity, whose noise response is sampling noise, fails calibration (R-squared 0.71 < 0.9). Second, a confounded calibration in which noise and blur co-vary passes the goodness-of-fit screen (R-squared 0.9999) yet mis-corrects by 9.5% when applied under noise alone—the screen cannot detect a confounded design, so descriptors must be calibrated one at a time. Third, applying a white-noise calibration to correlated noise of the same magnitude fails catastrophically (264% error): misspecification outside the calibrated regime is not caught by the acceptance rule, which motivates the out-of-sample validation reported here as a required companion to the rule. Finally, relating the calibration R-squared to the held-out remaining drift across all 75 features with a measurable noise response (Figure 6a) shows the expected monotone trend—higher calibration fit predicts smaller remaining drift, with a median 18% of drift remaining across accepted features—an empirical, out-of-sample grounding for the acceptance threshold beyond goodness of fit.

## 4. Discussion

### 4.1. Principal Findings

The framework demonstrates that a fully synthetic, patient-data-free testbed can reproduce the qualitative behaviour that motivates radiomics harmonisation—features drifting under blur, noise, slice profile, and voxel size—while retaining exact ground truth and full shareability. Three observations follow. First, an independently implemented feature core that reproduces all 482 IBSI digital-phantom benchmark values gives a trustworthy measurement instrument, so that any instability observed downstream is attributable to the simulated acquisition rather than to implementation drift; residual disagreement among standards-aligned toolkits [6] is a known confounder that a benchmarked core removes. Second, the stability atlas shows that, under a deliberately harsh acquisition envelope, most features are poorly reproducible (median ICC 0.13, 95% CI 0.03–0.19, with a median 77% of per-feature variance attributable to the acquisition condition and under 1% to stochastic realisation), and it exposes a subtle failure mode: features that are constant by construction produce an ICC of 1.0 that is a definitional artefact rather than evidence of robustness; the revised atlas therefore excludes such features from estimation entirely, a guard any reliability screen should adopt. Third, the normalisation result is a proof of concept, not a general solution. It is exact where the physics is known and invertible (intensity variance under additive noise), extends—as a physics-informed, empirically calibrated variant—to at least one higher-order texture feature (GLCM contrast, corrected to within 5% on held-out data), and, importantly, refuses features whose response the model cannot describe rather than correcting them blindly—an explicit, auditable failure that contrasts with black-box harmonisation.

### 4.2. Relation to Existing Tools

The framework’s individual ingredients—synthetic phantoms, IBSI benchmark validation, acquisition sweeps, analytic noise correction—each have precedents, and its contribution should be judged as a combination. Feature extraction toolkits such as PyRadiomics [5] implement standardised feature definitions on user-supplied images; they neither generate ground-truth phantoms nor simulate acquisition, and cross-toolkit agreement itself remains imperfect [6]. The IBSI digital phantom and benchmark tables [4] are a fixed validation reference—indispensable for verifying an implementation, but not configurable and not designed for stability screening. Physical texture phantoms such as the credence cartridge used in multi-scanner studies [3] capture real scanner physics, but their texture is not parametrically controllable, their ground truth is not analytic, and the scans themselves are not always shareable. Anthropomorphic computational phantoms and scanner-specific projection-domain simulators (for example XCAT and DukeSim [9,10]) model the imaging chain with far higher physical fidelity, including reconstruction; they are correspondingly heavier to run and configure, and are aimed at dosimetry and image quality research rather than exhaustive per-feature reproducibility screening. Cohort-based harmonisation such as ComBat [7,8] adjusts feature distributions statistically after the fact, requiring a reference cohort and offering no per-feature physical model or refusal mechanism. Against this landscape, radiomics-phantom deliberately trades physical fidelity for control, shareability, determinism, and coverage: parametric ground truth, a fully benchmarked independent feature core, a seeded acquisition sweep, reliability estimates with uncertainty, and an auditable normalisation that declines what it cannot model, all in one dependency-light open package. The capabilities that are unavailable in the prior systems are not the individual stages but their closed loop: the same code generates the texture, degrades it, measures it, rates every feature, and either corrects a feature or refuses to.

### 4.3. Limitations

The main limitations follow from the synthetic design. Gaussian-random-field texture is a controlled idealisation, not a substitute for the full complexity of real tissue; real parenchyma exhibits non-Gaussian, non-stationary, multi-scale structure that no stationary random field reproduces. The acquisition simulator operates strictly in the image domain, and this bounds what the atlas can claim. Because degradation is applied to the reconstructed image rather than simulated through projection, several classes of effects that shape real CT radiomics are absent: beam hardening and streak artefacts (non-stationary, object-dependent bias); the non-uniform, anisotropic noise power spectrum imposed by filtered back-projection or iterative reconstruction kernels (the simulator’s correlated noise is stationary, with an isotropic in-plane spectrum); nonlinear iterative and deep learning reconstruction, whose noise texture depends on the signal; tube-current modulation, which makes dose and, hence, noise spatially varying; scatter and photon starvation; partial-volume behaviour at high-contrast interfaces; and detector- and geometry-specific effects, together with their interactions with patient size and positioning. These omissions have an asymmetric consequence for generalisability. A feature that appears stable under the simulator’s stationary, well-behaved degradations may still be destabilised by non-stationary artefacts in clinical images, so positive (stable) findings in this atlas must not be transferred to clinical claims without external validation. Conversely, a feature that is already unstable under these mild degradations is unlikely to become stable under harsher real physics, so the atlas’s negative findings—which are the large majority—are the more transferable ones. Closing the gap requires calibrating the simulator against physical-phantom scans or public multi-scanner datasets, and coupling to projection-domain simulation chains [9,10]; both are planned (Section 4.5).

The stability atlas, though expanded in this revision to fifteen targets and nine conditions with bootstrap confidence intervals, remains one chosen envelope. Conclusions about a specific feature’s robustness are conditional on the swept textures and conditions, and the confidence intervals quantify sampling uncertainty within the design, not uncertainty about the design. The normalisation is demonstrated for two feature–response pairs under a single descriptor whose true value is known to the simulator; in practice, the noise level must be estimated from images, responses may be signal-dependent, spatially correlated, or multi-descriptor, and many features will have non-invertible or non-monotonic responses. The $R^{2}\geq 0.9$ acceptance threshold is a pragmatic screen rather than a calibrated statistical test; complementing it with out-of-sample error bounds and misspecification diagnostics is future work. No clinical, regulatory, or real data validation is claimed. As released, radiomics-phantom is a research reference, not a clinical or regulatory-grade tool.

### 4.4. Computational Cost

The framework is light enough to run interactively on commodity hardware, which is part of its intended role as a screening testbed. On a single CPU core of a containerised Linux environment (Python 3.11), generating one $32^{3}$ phantom takes about 3 ms, simulating one acquisition about 2 ms, and extracting the complete 136-feature vector about 80 ms. The full expanded atlas—15 targets × 9 conditions = 135 simulated acquisitions with feature extraction, followed by 2000 bootstrap replicates over 134 features—completes in roughly half a minute, and the entire set of results and figures in this paper regenerates in under a minute. Cost scales linearly in the number of (target, condition) cells and roughly with voxel count for generation and simulation; texture-matrix features dominate the extraction time. Timings are machine-dependent and are therefore written to a separate file (paper/figures/timing.json) rather than asserted by the test suite.

### 4.5. Future Work

Planned work proceeds along five lines. (1) External calibration and validation: calibrating the simulator’s noise magnitude, noise texture, and resolution against physical-phantom scans (for example the credence cartridge [3]) and public multi-scanner datasets, and quantifying agreement between synthetic and real acquisition-induced feature drift across scanners, kernels, doses, and slice thicknesses. (2) Cross-toolkit verification: running the atlas’s feature vector through PyRadiomics and other IBSI-aligned implementations under identical preprocessing to bound implementation-induced differences beyond the IBSI benchmark values. (3) Richer acquisition physics: projection-domain coupling [9,10], non-stationary and signal-dependent noise, reconstruction-kernel-specific noise power spectra, and interactions among parameters. (4) Broader designs: larger factorial sweeps over texture parameters (including lesion size, contrast, and intensity distribution), sensitivity analyses over the design itself, and segmentation–perturbation experiments. (5) Normalisation at scale: per-feature response libraries over multiple descriptors, noise-level estimation from images, uncertainty-aware correction with out-of-sample error bounds, and explicit tests of model misspecification. Each line extends the released, tested core rather than replacing it.

## 5. Conclusions

We present an open, fully synthetic framework for studying radiomics feature stability without any patient data, combining a deterministic 3D texture-phantom generator, an IBSI-aligned feature core validated against all 482 published digital-phantom benchmark values, an image-domain acquisition simulator, a per-feature stability atlas based on ICC(2,1) with bootstrap 95% confidence intervals, and a proof-of-concept physics-based normalisation that corrects features (demonstrated on intensity variance and GLCM contrast) from their known or calibrated acquisition responses and refuses features it cannot model. Because the study is fully synthetic, it is completely shareable and, within a pinned software environment, exactly reproducible. The contribution is a reproducible, patient-data-free testbed for radiomics reproducibility research and a transparent, auditable complement to cohort-based harmonisation for specific cases where the underlying physics is known or empirically invertible.

## Data Availability

The original data presented in this study are openly available. All code, the phantom generator, the IBSI feature core, evaluation scripts, and the test suite are available in the radiomics-phantom repository on GitHub at https://github.com/Institute-of-One/radiomics-phantom (accessed on 17 August 2026) under the MIT licence, and are archived on Zenodo at https://doi.org/10.5281/zenodo.21309874 (concept DOI, all versions; accessed on 17 August 2026). All data analysed are synthetic and produced by the included reproducible generator; no patient, clinical, or third-party data were used. The only external reference data are the public IBSI digital phantom and its published benchmark values.

## Supplementary Materials

The following supporting information can be downloaded at: https://www.mdpi.com/article/10.3390/jimaging12080392/s1, File S1: feature_inventory (mapping of all 482 IBSI benchmark values to atlas inclusion); File S2: stability_atlas (per-feature ICC(2,1) with realisation-aware bootstrap 95% confidence intervals, ICC(3,1), leave-one-condition-out range, and four-component hierarchical variance-decomposition fractions; constant features are listed separately as excluded from estimation).

## Abbreviations

The following abbreviations are used in this manuscript:

IBSI	Image Biomarker Standardization Initiative
ICC	Intraclass Correlation Coefficient
CCC	Concordance Correlation Coefficient
CI	Confidence Interval
GLCM	Grey-Level Co-occurrence Matrix
CT	Computed Tomography
HU	Hounsfield Unit
GenAI	Generative Artificial Intelligence
